# The Vault Nanoparticle: A Gigantic Ribonucleoprotein Assembly Involved in Diverse Physiological and Pathological Phenomena and an Ideal Nanovector for Drug Delivery and Therapy

**DOI:** 10.3390/cancers13040707

**Published:** 2021-02-09

**Authors:** Gianni Frascotti, Elisabetta Galbiati, Matteo Mazzucchelli, Maria Pozzi, Lucia Salvioni, Jacopo Vertemara, Paolo Tortora

**Affiliations:** Department of Biotechnology and Biosciences, University of Milano-Bicocca, 20126 Milano, Italy; gianni.frascotti@unimib.it (G.F.); elisabetta.galbiati@unimib.it (E.G.); matteo.mazzucchelli@unimib.it (M.M.); m.pozzi42@campus.unimib.it (M.P.); lucia.salvioni@unimib.it (L.S.); jacopo.vertemara@unimib.it (J.V.)

**Keywords:** vault nanoparticle, major vault protein (MVP), multidrug resistance (MDR), nanomedicine

## Abstract

**Simple Summary:**

In recent decades, a molecular complex referred to as vault nanoparticle has attracted much attention by the scientific community, due to its unique properties. At the molecular scale, it is a huge assembly consisting of 78 97-kDa polypeptide chains enclosing an internal cavity, wherein enzymes involved in DNA integrity maintenance and some small noncoding RNAs are accommodated. Basically, two reasons justify this interest. On the one hand, this complex represents an ideal tool for the targeted delivery of drugs, provided it is suitably engineered, either chemically or genetically; on the other hand, it has been shown to be involved in several cellular pathways and mechanisms that most often result in multidrug resistance. It is therefore expected that a better understanding of the physiological roles of this ribonucleoproteic complex may help develop new therapeutic strategies capable of coping with cancer progression. Here, we provide a comprehensive review of the current knowledge.

**Abstract:**

The vault nanoparticle is a eukaryotic ribonucleoprotein complex consisting of 78 individual 97 kDa-“major vault protein” (MVP) molecules that form two symmetrical, cup-shaped, hollow halves. It has a huge size (72.5 × 41 × 41 nm) and an internal cavity, wherein the vault poly(ADP-ribose) polymerase (vPARP), telomerase-associated protein-1 (TEP1), and some small untranslated RNAs are accommodated. Plenty of literature reports on the biological role(s) of this nanocomplex, as well as its involvement in diseases, mostly oncological ones. Nevertheless, much has still to be understood as to how vault participates in normal and pathological mechanisms. In this comprehensive review, current understanding of its biological roles is discussed. By different mechanisms, vault’s individual components are involved in major cellular phenomena, which result in protection against cellular stresses, such as DNA-damaging agents, irradiation, hypoxia, hyperosmotic, and oxidative conditions. These diverse cellular functions are accomplished by different mechanisms, mainly gene expression reprogramming, activation of proliferative/prosurvival signaling pathways, export from the nucleus of DNA-damaging drugs, and import of specific proteins. The cellular functions of this nanocomplex may also result in the onset of pathological conditions, mainly (but not exclusively) tumor proliferation and multidrug resistance. The current understanding of its biological roles in physiological and pathological processes should also provide new hints to extend the scope of its exploitation as a nanocarrier for drug delivery.

## 1. Introduction

The vault nanoparticle was first discovered in 1986 as a contaminant of rat liver coated vesicles [1]. In the following years, plenty of investigations provided detailed structural characterizations and led to the identification of its molecular components. It was dubbed vault as its morphology is reminiscent of the ceilings of ancient cathedrals. It was established, in particular, that its prevailing component is the 97 kDa major vault protein (MVP) [2], which is present in 78 copies in each complex. The MVP assembly generates a barrel-like structure enclosing a large internal cavity, wherein some small noncoding RNAs [3,4,5] and other protein species, i.e., the 193 kDa vault poly(ADP-ribose) polymerase (vPARP) [6] and the 290 kDa telomerase-associated protein-1 (TEP1) [7], are accommodated. It should be noted, however, that for the most part the molecular components in the vault cavity are also non-vault-associated and found in different cellular locations, as further detailed below [8,9].

**Figure 1:**
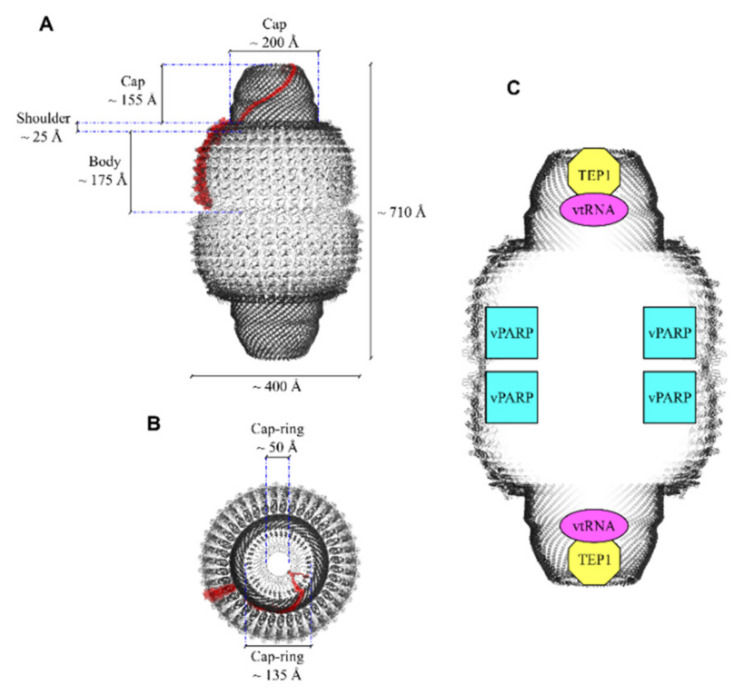
Structural features of the vault nanoparticle. (**A**) Side view, where a single major vault protein (MVP) molecule is colored in red. (**B**) Top view. (**C**) Cross-section including a schematic representation and location of the minor vault molecular components.

The envelope is formed by two symmetrical cup-like halves consisting of 39 MVP copies, each half displaying a bulging cap at the top and generating an invaginating waist where they contact each other. N- and C-termini of the MVP are located at the waist and the caps, respectively [10], the former being inside the vault cavity, the latter facing out (Figure 1). From the N-terminus to the C-terminus, each MVP monomer presents: a body region containing nine antiparallel β-sheet repeat domains (referred to as R1–R9); a shoulder region consisting of a single domain formed by four α-helices and a four-stranded β-sheet; a 155-residue cap-helix domain; a cap-ring domain [11,12,13]. Overall, the molecular mass of the nanoparticle is about 13 MDa and the size 72.5 × 41 × 41 nm. The minimum vault structure, endowed with its natural morphology, can be produced by expression of the sole MVP in insect cells [14,15]. More recently, recombinant vault nanoparticles were produced in the methylotropic yeast *Pichia pastoris* and were proven to be morphologically indistinguishable from those produced in insect cells [16]. A singular trait of the vault nanoparticle is the way it assembles, as it was shown that biosynthesis and assembling of MVP subunits take place sequentially at the level of polyribosomes, which therefore act similar to a 3D nanoprinter [15]. It is also worth mentioning its dynamic nature, as it can exchange individual MVP subunits in vivo, as well as separate at the particle waist and reassemble, thus reconstituting the whole vault. In this way, they can deliver molecules stored in their interior or take up molecules from the medium, which may be relevant to their physiological functions [17].

Although not ubiquitous, vaults are highly conserved and widespread among animal and possibly plant organisms [18,19] and it is estimated that there are 10^4^ to 10^5^ particles per cell [4,20]. Nevertheless, their physiological roles and involvement in pathology remain only partially understood, despite extensive investigations being carried out in recent decades. Here, we present a comprehensive review, mainly focusing on the current knowledge of their roles in major cellular phenomena, such as signal transduction, control of gene expression, apoptosis, as well as in their dysregulation and the resulting onset of pathological conditions. 

## 2. The Minor Components of the Vault Nanoparticle

### 2.1. Poly(ADP-Ribose) Polymerase

Poly(ADP-ribose) polymerases (PARPs) form a family of enzymes that, when sensing DNA breaks, use NAD^+^ as a substrate to catalyze the addition of several ADP-ribose moieties to acidic residues of a set of target proteins, with concurrent release of free nicotinamide. Linear or branched chains are thus formed. Such enzymes are involved in a number of key cellular processes mostly ensuring genome stability, such as DNA repair, but also replication, transcription, modulation of chromatin structure, and apoptosis. There is some evidence that PARPs can also effect automodification. Plausibly, ADP-ribosylation modulates the activity of target proteins [21,22]. 

The PARP family includes at least 18 members, among which PARP-1 may be regarded as the prototypic one [23]. It consists of three main domains: (1) the N-terminal DNA-binding domain (DBD), containing two zinc fingers and a nuclear localization sequence (NLS); (2) the automodification domain, containing a breast cancer susceptibility protein C-terminus motif (BRCT), which is common in many DNA repair and cell cycle proteins, and is involved in protein–protein interactions; (3) the highly conserved C-terminal catalytic domain. The 1724-residue-long vPARP, also referred to as PARP-4, has no DBD. Instead, it displays a BRCA1 C-terminal (BRCT) domain at the N-terminus, followed by the catalytic domain, a vault inter-alpha-trypsin (VIT) domain, a vWa von Willebrand type a (vWa) domain, an NLS and C-terminal, and 162-residue-long INT domain, which is capable of specifically binding to MVP with 0.2–0.3 µM affinity, close to the waist of the nanoparticle and in its interior [24]. BRCT domains are generally found in proteins involved in cell cycle regulation and/or DNA repair [25,26]; VIT and vWA domains are structurally related [27,28]. In particular, the vWA domains are endowed with a Rossman fold structure and participate in metal binding, in multiprotein complexes, and in proteins that mediate cell adhesion [9,28].

The estimates of vPARP copy number per vault are somewhat divergent [9,29,30]. The most reliable figure is probably around nine [30], but it cannot be excluded that it may vary depending on the physiological conditions, in keeping with the observations that vPARP is only partially associated with vaults and is also found in the nucleus, in the cytoplasm, and in the mitotic spindle during mitosis [6,31]. This suggests a possible exchange between free and vault-associated vPARP. It was also observed that cytoplasmic vPARP polymerizes into rods whose lengths seems to be inversely correlated with MVP expression levels [31]. Finally, evidence was also provided that, at least in vitro, vPARP ADP-ribosylates itself and MVP [6].

### 2.2. Telomerase-Associated Protein-1 and Untranslated RNAs

Telomerases are multi-sub-unit proteins that also contain RNA molecules used as a template to ensure telomere length maintenance via their reverse transcriptase activities. Telomerase activity was first identified in the ciliate Tetrahymena in a complex containing two protein components, i.e., p80 and p95, as well as an RNA template [32]. Telomerase-Associated Protein-1 (TEP1), identified through its N-terminal region homologous to Tetrahymena p80, was shown to be a subunit of both vault and mammalian telomerases [7,33,34]. In the telomerase complex, reverse transcriptase (hTERT) is the catalytic subunit [35], whereas TEP1 is catalytically inactive [7,36]; its deletion does not affect telomerase activity or telomere length, as shown in a TEP1-deficient mouse model [37,38]. In vaults, TEP1 binds to specific vault-associated RNA molecules (vtRNAs), as shown by a yeast three-hybrid assay [7,39]. Cryo-EM confirmed that TEP1, as well as vtRNAs, bind the interior of vaults at the level of their caps [36]. As vtRNAs are not found in vaults from TEP1-deleted mouse animal models, this confirms a direct TEP1/vtRNA interaction [36,40]. However, unlike vPARP, no TEP1-binding site has been found in MVP by a yeast-two hybrid screening [41], suggesting that this site constitutes more than one MVP molecule [39,42], with 2–4 TEP1 copies being bound to each vault [9]. TEP1 is a 240 kDa protein that has four 30-residue N-terminal repeats, whose role is still unclear, followed by the Telomerase, Ro and Vault (TROVE) domain, which is evolutionarily conserved and is responsible for RNA binding [43]. The only other protein containing the TROVE domain is Ro, which is found in both prokaryotes and eukaryotes [44]. Ro binds to Y RNAs, other noncoding RNAs resembling vtRNAs, and is involved in diverse RNA processing and quality control events [45]. Molecular modeling of the TROVE domain based on the known *Xenopus laevis* Ro60 protein predicted a toroidal-shaped RNA-binding module, consisting of HEAT repeats [45,46]. The TROVE domain is followed by a vWA domain, also found in vPARP, as above outlined [39,43]. In TEP1, it may be in some way involved in RNA binding, as its removal affects TROVE/vtRNA interaction [39]. Downstream of the vWA domain, a NACHT-type NTPase domain is located [47], which has been suggested to regulate RNA binding [39]. Then, close to the C-terminus, there are 21 putative WD (tryptophan-aspartate) repeats that probably fold as two connected seven-bladed propellers [32,42] and might be involved in the interaction with other molecular partners [32,48]. Although the physiological roles of TEP1 still await full elucidation, it is assumed that it might be involved in telomere maintenance, as suggested by the fact that disruption of the p80/p95 complex in Tetrahymena results in telomere lengthening [39].

Noncoding RNAs have been long implicated in post-transcriptional gene silencing. However, in more recent times they have also emerged as key players in gene expression and genomic stability regulation at the transcriptional level [49]. Specifically, vtRNAs are small, noncoding RNAs, 80–140 nt in size, quite likely evolutionarily related to tRNAs in that both RNA types are transcribed by RNA polymerase III, and the encoding genes contain a split promoter consisting of the so-called A box and B box elements internal to the transcription unit [50,51,52]. Akin to the minor vault proteins, vtRNAs are largely not associated with the nanoparticle, the bound fraction representing about 5% of the total vault mass, which suggests that they are involved in diverse cellular interactions [4]. In the human genome, four vtRNAs are encoded on chromosome 5q31 in two different loci. At the VTRNA1 locus, three vtRNAs are encoded, i.e., vtRNA1-1, vtRNA1-2 and vtRNA1-3, whereas at the VTRNA2 locus, the sole vtRNA 2-1 (formerly referred to as pre-miR-886 or CBL3) is encoded. Furthermore, two vtRNA pseudogenes are located on chromosomes 2 and X [50,51]. Generally, vtRNA1-1 is the most represented vtRNA variant, although diverse vtRNA expression levels are detected in different tissues. For instance, high vtRNA1-3 content in the vaults is associated with multidrug resistance [3,5]. Sequence comparison highlights only a limited conservation among the four vtRNA types, as well as among species [50]. Regarding their structures, prediction methods assign both paired and nonpaired regions, although their precise tertiary fold has still to be determined [3,50,53]. However, a mutational analysis suggests that mouse vtRNA binds preferentially to TEP1 when the central loop region is single stranded [39].

In the following chapters, the current knowledge regarding the involvement of vault proteins and vtRNAs in physiological and pathological processes will be presented in detail.

## 3. The Involvement of the Vault Nanoparticle in Normal and Pathological Processes 

### 3.1. How the Vault Nanoparticle Participates in Diverse Cellular Regulatory Mechanisms and Pathways

In the last two decades, extensive investigations on the vault nanoparticle have revealed an overwhelmingly complex picture of its involvement in normal and pathological processes. In the present paragraph, current knowledge will be discussed, also keeping in mind that, to date, many issues still require clarification. The information presented here deals with vault’s involvement in regulatory mechanisms and the relevant interactors, summarized in Table 1. 

After its discovery in 1986 [1], much effort was put into clarifying the roles of this molecular assembly. Initially, a major clue came from its identification as the lung resistance protein (LRP) [80], along with plenty of following papers which highlighted its involvement in multidrug resistance (MDR). Subsequent investigations revealed that it participates in a multiplicity of cellular pathways and phenomena.

In line with its well-known prosurvival role, one of the earliest findings was its association with estrogen receptor in nuclei of MCF-7 breast cancer cells [54]. It was suggested that the vault nanoparticle might mediate the nuclear transport of the receptor molecule, although the molecular details of the process were not investigated. 

One major pathway boosted by MVP is the epidermal growth factor (EGF)/phosphatidyl-inositol-3-kinase (PI3K)/protein kinase c (AKT) signaling axis. It was shown that in human glioblastoma (GBM) cells, MVP is upregulated (albeit by unknown mechanisms), which is paralleled by activation of migratory and invasive potential [55,81]. Actually, different reports provide evidence of MVP-mediated, phosphorylation-dependent activation of AKT, which promotes cell survival, growth, proliferation, and prevents apoptosis [64,82]. Furthermore, phosphatase and tensin homolog (PTEN), which prevents AKT activation through dephosphorylation of the AKT activator PIP3, directly binds to MVP [56] via two putative NLSs, thus undergoing MVP-mediated importation into the nucleus [83], although other import mechanisms may also be acting [84]. The binding is also Ca^2+^-dependent and nuclear PTEN localization is decreased by increasing concentrations of the cation [85]. As nuclear PTEN induces G1 cell cycle arrest, whereas cytoplasmic PTEN induces apoptosis [86], this suggests that MVP-mediated nuclear-cytoplasmic PTEN partitioning might be one important factor accounting for the antiapoptotic and protumorigenic role of MVP, also linked to Ca^2+^-dependent regulation of the cell cycle.

Another molecule potentially capable of modulating vault’s action in this signaling pathway is PRKR-like endoplasmic reticulum kinase (PERK), which is involved in one endoplasmic reticulum (ER) stress-signaling pathway [87]. Following unfolded protein response and/or tumor-related hypoxia conditions, PERK undergoes autophosphorylation, which triggers PERK-dependent phosphorylation of the translation initiation factor eIF2α. This results in protein synthesis inhibition, which involves a survival advantage for tumor cells. As far as the PI3K/AKT signaling pathway is concerned, it was shown that inactive PERK interacts with the vault complex, which prevents vault-mediated PTEN nuclear transport, possibly downregulating the pathway [57,87]. Although the details and the physiological significance of this mechanism still await better understanding, it points, nevertheless, to a regulatory link between ER stress and the signaling pathway.

A further vault interactor, which plays a major role in key cellular events is Bcl2-associated athanogene 3 (BAG3) [58]. This molecule belongs to the family of the BAG co-chaperones, acts as a nucleotide exchange factor of Hsc/Hsp70, and is also capable of interacting with a multiplicity of other molecular partners, thus modulating several cellular functions. It is a well-known antiapoptotic agent, but during cellular aging or under stress it also stimulates autophagy [88]. In line with its antiapoptotic role, it has been implicated in resistance to chemotherapy in small cell lung cancer (SCLC) cells [89]. Adriamycin (brand name of doxorubicin, DOX), a senescence-inducing drug, triggered BAG3 upregulation in MCF7 breast cancer cells. Additionally, this cochaperone interacted with MVP irrespective of drug administration, but to a larger extent in its presence, the interaction leading to MVP stabilization and translocation to the nucleus [58]. Furthermore, silencing of either protein switched the response to the drug to apoptosis in several breast cancer cell lines. Most notably, this was accompanied by a decrease in Extracellular signal Regulated Kinase 1/2 (ERK1/2) pathway activation, as documented by a significant reduction in its phosphorylated form (pERK1/2). ERK1/2, a well-known Mitogen-Activated Protein Kinase (MAPK) is under control of a wide repertoire of receptor tyrosine kinases (RTKs), including several growth factors [90], which regulate cell proliferation. Overall, these results have suggested a model whereby BAG3 mediates MVP accumulation in the nucleus in association with ERK1/2, which results in activation of the latter and the ensuing prosurvival action. According to the model, the balance between senescence and apoptosis would be regulated by BAG3 levels [58]. Relevant to this mechanism of gene expression control, it was found that in COS-7 cells, MVP also interacted with YPEL4, a member of the widespread YPEL family, which contains zinc-finger-like metal-binding domains [59]. YPEL4 is known to stimulate the transcriptional activator Elk-1 in the MAPK signaling pathway, and its direct interaction with MVP resulted in substantial reduction in the transcriptional activity [59]. Yet, the regulatory significance of this interaction awaits further investigations.

MVP was also shown to interact with the tumor-promoting B7-H3 glycoprotein [60]. B7 immunomodulatory proteins bind to members of the CD28/CTLA-4 family, which act as the costimulatory signal in the activation of T cells. B7-H3 is found as both a transmembrane and a soluble form [91]. In immortalized human mammary epithelial cells (HMLEs), B7-H3 overexpression resulted in a dramatic increase in cancer stem cell populations, a condition enhancing drug resistance, metastasis, and relapse. This was paralleled by MEK phosphorylation and its resulting activation. MEK is a key component of the Ras/Raf/MEK/ERK cascade reaction, a pivotal signaling pathway in MAPKs, which is under control of several extracellular hormonal stimuli [92]. By coimmunoprecipitation and mass spectrometry analysis, MVP was shown to interact with B7-H3 and the interaction was substantially weaker with a truncated form of the latter, lacking the cytosolic domain. Furthermore, MEK phosphorylation and the resulting activation was significantly reduced in MVP-depleted cells. Taken together, these results lead to the conclusion that B7-H3, in association with MVP, is capable of enhancing B-RAF/MEK interaction and the ensuing MEK activation. In keeping with his conclusion, depletion of MVP significantly increased the nonstem cell population [60].

Another MVP interactor is the Src homology 2 (SH2) domain-containing tyrosine phosphatase (SHP-2) [61], which in recent years has emerged as a major regulatory protein of RTK and cytokine receptor signaling [93]. SHP2 interacts with tyrosine phosphorylated MVP in a substrate-trapping manner—i.e., without the involvement of phosphatase activity. Whereas a basal level of MVP phosphorylation was also present in the absence of hormonal stimulation, it was boosted by EGF and led to the transient formation of a ternary complex, MVP/SHP2/pERK. Although MVP only subtly affected ERK activation as shown in MVP-deficient mouse embryo fibroblasts (MEFs), Elk-1 downstream activation was instead significantly impaired in the same cells. In line with this observation, they also displayed significantly increased cell death as compared with the controls. Overall, these findings further highlight the prosurvival role of MVP and suggest it might function as a scaffold protein for both SHP-2 and ERK. In the proposed model, SHP-2 might modulate the level of MVP phosphorylation, thus playing a role in cell survival signaling [61].

These findings also point to MVP as a target of protein tyrosine kinase(s), which has raised the obvious issue of its/their identification. Actually, several putative MVP phosphorylation sites have been long identified, along with in vitro MVP phosphorylation by AKT and casein kinase II from electric ray [94]. Experiments subsequently carried out in cell-free extracts from mammalian cell lines have also detected MVP phosphorylation [95]. However, neither conclusive evidence supporting the physiological significance of these observations, nor a clear-cut identification of in vivo-acting kinases was provided by these studies. This was instead achieved in a more recent paper [62], whereby the authors searched for proteins interacting with the SH2 domain of the Src tyrosine kinase by taking advantage of a pull-down approach followed by proteomic analysis. Src is involved in several signaling pathways and controls a wide range of diverse cellular functions, particularly cell proliferation and differentiation [96]. They thus identified MVP as a Src interactor in human stomach tissue and in stomach cancer cells and showed that EGF stimulation triggers Src-dependent MVP phosphorylation, translocation from the nucleus to the cytosol, and colocalization at the perinuclear region [62]. It may not be straightforward to disentangle the network of causal relationships that link the functions of SHP-2 phosphatase and Src kinase. It is conceivable, however, that Src-catalyzed phosphorylation of MVP may represent a prerequisite for the formation of the MVP/SHP2/pERK ternary complex.

Another MVP interactor recently identified, which might play a major role in modulating its prosurvival action, is the ε isoform of 14-3-3, a wide family of highly conserved cellular proteins that regulate several pathways. They have been implicated in cancer development, which is well-justified by their involvement in mitogenic and cell survival signaling, cell cycle control and apoptotic cell death [97]. In particular, the role of 14-3-3ε, the most conserved member of the family, was investigated in hepatocellular carcinoma (HCC) where this isoform is upregulated. HCC cells stably expressing 14-3-3ε underwent apoptosis when treated with bleomycin, an anticancer drug capable of inducing double-strand DNA breaks [98]. Concurrently, by taking advantage of a proteomic approach, it was shown that MVP underwent bleomycin-dependent phosphorylation at the Thr52 residue and that dimeric 14-3-3ε bound to Thr52- and Ser864-phosphorylated MVP monomers, thus hindering normal vault particle assembly and impairing vault-sustained drug sequestration and its extracellular disposal [98]. It appears therefore that the interaction between these two molecular partners modulate MDR in HCC. 

Some of the regulatory mechanisms depicted above make it apparent that MVP is target of multiple phosphorylation events, involving both tyrosine (Src) and serine/threonine residues (14-3-3ε). Although not all phosphorylatable residues have been identified, these results suggest nevertheless that the vault nanoparticle is subject to a complex regulatory pattern, based on the modulation of such modifications.

STAT3, a transcription factor promoting cell survival/proliferation, motility, and immune tolerance [99], was also shown to be under MVP control in airway smooth muscle cells (SMCs). In fact, following stimulation by either the cytokine interleukin-22 (IL-22) or platelet-derived growth factor (PDGF), it underwent phosphorylation-dependent activation along with AKT, which was quite likely mediated by MVP, as knockdown of the latter substantially impaired the process. Concomitantly, IL-22 stimulation was also paralleled by MVP S-glutathionylation, as supported by mass spectrometry data. MVP was also subject to an equilibrium between S-glutathionylated and deglutathionylated states, the latter form being generated by the enzymes glutaredoxin-1 and thioredoxin. When S-glutathionylated, MVP could sequester myosin-9, a protein whose functions are otherwise not well-understood, and which acted as a proapoptotic factor when not bound to MVP. Thus, MVP S-glutathionylation prevented the apoptotic process [64]. In such an intricate regulatory network, MVP is involved in STAT3 and AKT activation, which confirms its prosurvival role, but it also exerts a direct antiapoptotic effect, probably under control of the cellular redox status. A major achievement of this contribution is also the discovery of a further reversible post-translational modification (PTM) in addition to phosphorylation. As in the case of the several MVP phosphorylation events, this PTM is also suggestive of an additional regulatory level for the nanocomplex.

The vault nanoparticle has been implicated in other regulatory phenomena, whereby it modulates the activities of two E3 ubiquitin ligases—i.e., the constitutively photomorphogenic 1 (COP1), which is a RING finger ubiquitin ligase [65] and pVHL [66]. In mammalians, COP1 targets c-Jun for proteasomal degradation, one out of several members generating the combinatorial diversity of the heterodimeric AP-1 transcription factors. c-Jun is activated via phosphorylation by c-Jun N-terminal kinases (JNKs) in response to different stress stimuli, including UV irradiation. It is well-known that JNK belongs to the MAPK family and modulates the activity of several downstream proteins, thus controlling a multiplicity of cellular functions [100], including antistress and antiapoptotic effects [101]. As far as the role of COP1 is concerned, the mentioned investigation demonstrated association between COP1 and MVP in the cytosol of HEK293 cells by taking advantage of affinity purification and mass spectroscopy. Overall, a model was proposed envisaging that, under basal conditions, COP1 undergoes an as yet unidentified modification effected by a vault component (probably vPARP). Then, thanks to a free/bound equilibrium, COP1 would be released from vault, translocated into the nucleus, and bound to c-Jun, thus destining it for degradation. According to the model, following UV irradiation MVP would be instead tyrosine-phosphorylated, which would prevent it from interacting with COP1. As a result, unmodified COP1 would not bind and degrade c-Jun, which in turn, after undergoing JNK-catalyzed phosphorylation, would activate the transcription of genes under control of AP-1 [65]. The net outcome of this regulatory device is a UV-triggered, c-Jun-mediated transcriptional activation, whereby MVP acts as UV sensor and key player of the transcriptional switch.

MVP was also shown to modulate cellular adaptation to hypoxia. This phenomenon is mainly based on turnover regulation of Hypoxia-Inducible Factor-1α (HIF-1α) which, along with HIF-1β, forms the HIF heterodimeric transcription factor. This controls the transcription of a set of genes responsible for hypoxic adaptations. A specific proline hydroxylase (PHD2) hydroxylates HIF1α at two proline residues in an oxygen-dependent manner, which results in ubiquitination by the ubiquitin ligase von Hippel-Lindau protein (pVHL) and proteasomal degradation of HIF1α. In contrast, during hypoxia, the proline residues are not hydroxylated and pVHL cannot bind, which results in transcriptional activation [102]. MVP was clearly involved in the process, as its knockdown in human renal adenocarcinoma (ACHN) cells resulted in decreased HIF-1α ubiquitination. Concurrently, direct interaction of MVP with HIF-1α was demonstrated by coimmunoprecipitation. Evidence for possible recruitment of PHD2 and pVHL in the same complex was also achieved, although direct binding of these to MVP is not likely [66]. On the whole, these results indicate that MVP may in some way cause adaptation to hypoxic conditions, in keeping with its role in dealing with stressful conditions.

A further MVP interactor mainly acting in macrophages is class A scavenger receptor (SR-A), a cell-surface glycoprotein that mediates the uptake of several endogenous and exogenous substances, including oxidized low-density lipoprotein LDL. The uptake results in a set of downstream events, such as phagocytosis of bacteria, cell adhesion, and apoptosis. Additionally, macrophage conversion into foam cells ensues along with inflammatory cytokine overproduction, which has been implicated in the onset of atherosclerosis [63]. By immunoprecipitation coupled with mass spectroscopy, colocalization and cross-linking experiments, SR-A association with MVP at the membrane level and cytoplasm of mouse peritoneal macrophages was established [67]. Fucoidan, used as an SR-A ligand, triggered caveolin- but not clathrin-dependent endocytoses, which led to p38/JNK-mediated TNF-α production and MVP recruitment to lipid rafts. Furthermore, the essential role of MVP was proven by its knockdown causing a substantial dampening of the transduction process and, importantly, the inhibition of fucoidan-induced macrophage apoptosis [67]. It seems, therefore, that MVP may play a proapoptotic role in macrophages, unlike the well-established prosurvival role generally assigned to the nanocomplex. Thus, further investigations are required to clarify the physiological significance of this phenomenon.

When dealing with MVP interactors, it is worth mentioning that it was also identified as a novel target of caspases in human primary keratinocytes [68]. By mass spectroscopy and other complementary approaches, the authors showed that cells subjected to UVB irradiation experienced inflammasome activation, cytokine secretion, and caspase-1-dependent apoptosis. Concomitantly, MVP underwent caspase-1- and caspase-9-catalyzed cleavage at Asp441. A very similar pattern was observed in human primary fibroblasts following staurosporine-induced apoptosis while, in contrast, MVP was not cleaved in apoptotic THP-1 monocytic cell lines. Remarkably, a mutated (D441E), cleavage-resistant MVP variant overexpressed in epithelial cells subjected to apoptotic stimuli conferred protection against cell death [68]. Thus, MVP level control appears to be a key event in establishing the balance between apoptosis and cell proliferation, at least in the mentioned cell lines.

### 3.2. The Role of the Vault Nanoparticle in Virus Infection and Inflammation

Among the several pathways the vault nanoparticle was shown to be involved in, events related to virus infection and interferon (IFN) signaling have also been described, as reported in three papers from the same research group [69,70,71]. This group found high MVP expression in peripheral blood mononuclear cells (PBMCs), sera, and liver tissue from either hepatitis C virus (HCV)- or hepatitis B virus (HBV)-infected patients [69,70]. Regarding HCV, they demonstrated that two viral proteins (NS5A and Core protein) could activate MVP expression by binding the nuclear factor kappa B (NF-κB)- and specificity protein 1 (Sp1)-binding sites of the promoter (detailed in the next Section 3.4). Sp1 is a well-known constitutive transcription activator often involved in cell growth control and tumorigenesis [103]. In turn, MVP upregulation led to enhanced expression of a specific IFN regulatory factor isoform (IRF7), NF-κB activation, and translocation of both proteins to the nucleus, which eventually resulted in type-I IFN production [69]. Likewise, elevated MVP expressions in HBV-infected patients and hepatoma cell lines were also detected and a specific viral protein (HBx) was shown to stimulate MVP promoter activity. The authors then assessed possible MVP interaction with myeloid differentiation primary response 88 (MyD88), as this factor is recruited by most Toll-like receptors (TLRs) at the level of their cytoplasmic portion in the cascade that, following viral infection, leads to type-I IFN activation. They actually demonstrated that MVP participated in antiviral response as it bound to MyD88 via its middle domain, which resulted in NF-κB and IFN-β activation. However, unlike HCV, in the case of HBV infection two viral surface antigens also bound to MVP thus competing for the MVP/MyD88 interaction and preventing NF-κB and IFN-β signaling [70]. Irrespective of the diverse mechanisms of the antiviral responses, available data highlight a broad involvement of MVP in innate immune responses to viral infection, which also includes hepatitis vesicular stomatitis virus, influenza A virus, and enterovirus 71 [69], in keeping with its capability to generally cope with stressful conditions.

In a more recent report, it was also determined that forced MVP expression was sufficient to trigger hepatocellular carcinoma (HCC) in mice, which highlights a possible causal relationship between HBV or HCV infections and the onset of the cancer disease [71]. The key features of the underlying molecular mechanism were also identified, in that human double minute 2 (HDM2) E3 ubiquitin ligase (orthologous of the well-known murine variant MDM2) [104] formed reciprocally exclusive complexes with either IFN regulatory factor 2 (IRF2) or p53, and that MVP displaced HDM2 from IRF2, thus favoring HDM2 binding to p53 and the ensuing degradation of the latter. This mechanism was confirmed in mouse xenograft models, wherein both HBV and HCV infections resulted in MVP upregulation, HDM2-mediated p53 degradation, and carcinogenesis [71]. Interestingly, these findings may account for the mechanism by which hepatitis evolves into HCC.

MVP was also shown to be involved in a further proinflammatory responses to viral infection. In human lung epithelial A549 cells, this was triggered upon exposure to either influenza A virus or to a combination of poly(I:C) and IFN-γ to mimic the infection, and their ensuing binding to TLR3/RLR receptors [72]. Overall, this investigation determined a mechanism whereby MVP was upregulated by the treatments, which in turn resulted in potentiated IL-6 and IL-8 upregulations. This was sustained by MVP interaction with the transcription factors c-Fos, C/ERBβ-LAP and the NF-κB components p50 and p65, as shown by coimmunoprecipitation assays in human embryonic kidney HEK293T cells. Further, MVP promoted nuclear translocation of c-Fos and C/ERBβ-LAP, which eventually resulted in recruitment of NF-κB, c-Fos, and C/EBPβ to the relevant regions of IL-6 and IL-8 promoters and their transcriptional activation [72]. 

In a recent work, MVP was shown to also suppress NF-κB-mediated inflammation in macrophages [73]. The authors first observed MVP upregulation in some types of adipose tissue from high-fat-diet (obese) mice and human beings. Then, they demonstrated derangement of glucose and lipid metabolism and increased weight in obese MVP knockout mice compared with the wild-type littermates. Under suitable conditions, in MVP-deficient mice they also observed macrophage infiltration, increased proinflammatory cytokines in adipose tissue, and increased atherosclerotic lesions paralleled by an inflammatory response. How MVP deficiency results in such effects was clarified in MVP knockout mice macrophages subjected to LPS stimulation. The key finding was MVP colocalization with tumor necrosis factor (TNF) receptor-associated factor 6 (TRAF6), an E3 ligase playing a key role in NF-κB activation [105]. LPS stimulation triggers receptor oligomerization and recruitment of several proteins to the receptor cytosolic domain, including TRAF6, which also induces self-ubiquitination, as well as ubiquitination of other proteins of the receptorial complex. Through a sequence of events, this results in phosphorylation-dependent activation of IKK kinases, which in turn phosphorylate the NF-κB inhibitor I-κBα, thus destining it for degradation. This enables NF-κB translocation into the nucleus to initiate transcription. In contrast, MVP binding to TRAF6 prevented the cascade, thus suppressing inflammatory responses, which instead were exacerbated in MVP-depleted macrophages. Quite plausibly, MVP deficiency is responsible for the downstream effects, such as obesity, metabolic disorders, and atherosclerosis. Thus, the vault complex also stands out as a modulator of NF-κB-mediated proinflammatory response [73]. It is worthwhile noting the different roles MVP plays in modulating NF-κB activity in different physiological contexts. In the aforementioned case of HCV infection, it participated in a process eventually leading to NF-κB expression and activation of the relevant pathway in hepatoma cell lines [69], whereas MVP interaction with TRAF6 inhibited the same pathway in macrophages.

### 3.3. Regulatory Mechanisms under Control of vtRNAs

Overall, the current knowledge on how MVP is involved in major cellular processes highlights an astoundingly complex picture, whereby this nanocomplex stands out as a key regulator of several of them. Nevertheless, key regulatory roles for vtRNAs have also emerged, mainly in recent years.

The first report, whereby an interaction between vault-associated RNA and an intracellular protein was identified, provided evidence of a complex between the nucleic acid and the La autoantigen in HeLa cells [8]. However, the physiological significance of such an interaction has still to be clarified. More recently, further reports have identified vtRNAs’ involvement in novel interactions and regulatory roles, which implicates them in the control of transcriptional, developmental, autophagic, and apoptotic functions.

As far as macroautophagy is concerned, the vtRNA1-1 isoform was identified as a regulator of the process, as supported by its capability of binding sequestome-1/p62 [51,74], a selective autophagy receptor. The latter interacts via its UBA domain with ubiquitin chains attached to the autophagic cargo and via its LC3-interacting region (LIR) motif with ATG8 family proteins covalently attached to the inner membrane surface of the growing phagophore [106]. By using the RIP-qPCR technology, selective vtRNA1-1 binding to p62 was demonstrated in several human and murine cell lines, which resulted in autophagy inhibition. In turn, in HuH-7 cells vtRNA1-1 levels underwent starvation-induced decreases, irrespective of p62 levels, which resulted in more p62 being set free, with ensuing autophagy activation. Based on these observations, it was proposed that high vtRNA1-1 prevents autophagy by binding to p62, thus inhibiting its multimerization and the subsequent autophagosome assembly. It has still to be established whether the antiautophagic role vtRNA1-1 plays in the process may in some way involve other vault components [74]. It is worthwhile mentioning that, in this context, MVP also plays an antiautophagic role via the mammalian target of rapamycin (mTOR). This is an intracellular kinase that inhibits autophagy via phosphorylation of downstream effectors [107] and, in turn, is activated by AKT. Thus, MVP itself prevents autophagy when involved in the activation of the PI3K/AKT/mTOR pathway [55], although no direct interaction MVP/mTOR seems to take place.

vtRNA1-1 (but not other vtRNAs) was also shown to protect Burkitt lymphoma BL2 cells from Epstein–Barr virus (EBV)-induced apoptosis [75]. By individually expressing several latency phase III EBV proteins, it was shown that only latent membrane protein 1 (LMP1) significantly triggered vtRNA1-1 upregulation through two cytoplasmic C-terminal activator regions, and that the activating effect was mediated by both canonical and noncanonical NF-κB pathways. Significant enrichment of the dominant NF-κB member p65/RelA at the vtRNA1-1 promoter was detected by chromatin immunoprecipitation (ChIP) experiments in LMP1-overexpressing or EBV-infected BL2 cells. In turn, vtRNA1-1 upregulation conferred dosage-dependent apoptosis resistance by inhibiting both the extrinsic and the intrinsic pathways, irrespective of whether MVP was knocked down or not. However, the molecular interactions underlying the antiapoptotic action of vtRNA1-1 have still to be defined [75].

A recent paper has provided new and substantial evidence for an antiapoptotic effect caused by vtRNA1-1 [76]. By taking advantage of the CRISP/Cas9 technology, the authors generated knockout vtRNA1-1 and vtRNA1-3 HeLa cell lines. They thus demonstrated that the loss of vtRNA1-1 but not of vtRNA1-3 resulted in increased apoptosis under starvation conditions. Analysis of mRNA transcriptome was then performed by next generation deep sequencing, which highlighted significant reprogramming, including, in particular, activation of the PI3K/AKT pathway and the ERK1/2 MAPK pathways. These results were not anticipated given the prosurvival role of these pathways. Thus, these findings demand further investigations to establish the causal relationship between vtRNA1-1 knockout and the observed metabolic reprogramming, as well as its adaptive significance. Nevertheless, they clearly establish an antiapoptotic role for vtRNA1-1. Additionally, the specificity of this mechanism is noteworthy, in that vtRNA1-3 knockout did not produce any similar effect [76].

Recently, two other papers have identified mechanisms of vtRNAs being processed into smaller fragments referred to as svRNAs, which play regulatory roles in key cellular processes. Evidence in support of this was first achieved by deep sequencing experiments that identified different svRNAs in MCF7 cells [77]. In particular, it was shown that the 23 nt long variant svRNAb was derived from vtRNA1-1 and that Dicer, a component of the miRNA pathway, was involved in the processing. The authors also demonstrated that svRNAb, similar to miRNAs, could both guide sequence-specific cleavage of a complementary target RNA in vitro and bind to some Argonaute proteins, the core components of the RNA-induced silencing complex (RISC) [108]. Based on these findings, it was proposed that svRNAb shares mechanisms of gene expression regulation with miRNA. The authors also demonstrated that the CYP3A4 gene, which codes for cytochrome P450, was downregulated by svRNA, as shown by microarray and knockdown technologies. Cytochrome P450 is a well-known detoxifying enzyme that metabolizes many chemotherapeutic compounds [77]. Thus, these results suggest that their metabolism is under control of vtRNA1-1-dependent processing mechanisms.

Another major regulatory phenomenon modulated by vtRNA1-1 processing was described in human dermal fibroblasts [78]. It was observed that NSUN2, a m^5^C methyltransferase, methylated cytosine (C) 69 in vtRNA1-1 (in addition to several other coding and noncoding RNAs), as shown by RNA bisulfite sequencing on *NSUN2*^–^*/*^–^ human dermal fibroblasts after NSUN2 re-expression. Methylation enhanced vtRNA1-1 processing into different small, noncoding fragments, including svRNA4, the latter resulting from the cleavage at C69. RNA pull-down SILAC technology was then used to identify RNA-binding proteins showing differential affinity to methylated or nonmethylated vtRNA1-1. This led to the identification of the serine/arginine-rich splicing factor 2 (SRSF2), which actually bound with higher affinity to the nonmethylated form, thus sequestering it. In turn, svRNA4 apparently prevented the epidermal differentiation program, probably through post-transcriptional silencing, as supported by the failure of svRNA4-transduced keratinocytes to differentiate into a stratified squamous epithelium. Thus, the balance between nonmethylated vtRNA1-1 binding by SRSF2 and NSUN2-catalyzed methylation with the ensuing cleavage to yield svRNA4 dictates the fate of keratinocytes [78].

Additionally, unprocessed vtRNA1-1 could affect gene expression in MCF-7 cells, as shown in an investigation focused on the polypyrimidine tract-binding protein-associated splicing factor (PSF) [79]. This protein has two RNA-binding domains (RBDs) and a DNA-binding domain (DBD) and was originally identified as a protein component of spliceosomes [109]. In keeping with these features, it could bind to vtRNA1-1 at the level of the RBD, as shown by electrophoretic mobility shift assays. In vivo, RNA and chromatin immunoprecipitations demonstrated PSF interaction with vtRNA1-1 and the GAGE6 promoter, respectively. Additionally, transfection with vtRNA1-1 cDNA released PSF from the GAGE6 promoter region, showing that vtRNA1-1 and the promoter compete for PSF. Furthermore, the latter is a well-known transcriptional repressor of GAGE6 gene, whose expression confers chemoresistance on cancer [110]. In line with this scenario, MCF-7 transfection with vtRNA1-1 relieved transcriptional repression thus, enhancing DOX resistance, irrespective of the expression level of MVP [79].

Finally, a thorough investigation recently led to the identification of a vtRNA homolog in the kinetoplastid *Trypanosoma brucei*, which was referred to as TBsRNA-10 [45]. The identification was supported by a predicted secondary structure reminiscent of that of YRNAs and vtRNAs from several species, including bulged and paired tracts, as well as by its transcription being performed by RNA polymerase III. Additionally, coimmunoprecipitation experiments demonstrated its association with a TEP1 homolog. Importantly, an RNase H cleavage assay made it possible to identify different regions potentially capable of base pairing with other RNAs, as already observed in mammalian vtRNAs. *T. brucei* vtRNA is mostly located in a nuclear subdomain where it colocalizes with the ribonucleoproteic equipment required for *trans*-splicing (i.e., splicing of distinct RNA molecules), a process only observed in *T. brucei* and a few other organisms belonging to an early diverging branch of the eukaryotic tree. In the same subdomain, a small RNA element known as splice leader (SL), the key factor for *trans*-splicing, is also present. Furthermore, it was determined that *trans*-spliced mRNA production underwent a substantial decrease following vtRNA downregulation in a permeabilized cell system, which provides evidence that vtRNA/TEP1 is indeed required for the process. However, no direct interaction between either vtRNA or TEP1 with SL RNA could be detected, which points to some indirect effect. Thus, the understanding of the precise mechanism whereby the ribonucleoprotein complex participates in the process deserves further investigations [45]. These findings have raised the issue of whether higher eukaryotes may also possess similar mechanisms [111].

The picture provided by the above survey of vtRNA’s known functions is no less complex than that observed for MVP itself. Remarkably, vtRNAs can apparently fulfil their actions by both interfering with major cellular processes, such as autophagy and apoptosis, and modulating gene expression in several ways. In some cases, vtRNAs or their cleavage products may act as miRNAs, thus effecting gene silencing, but also a direct modulation of gene expression at the level of the promoter detected, as in the case of PSF [79]. Although in some reports their ultimate effect is not obvious, it seems that vtRNAs mostly foster prosurvival and drug resistance mechanisms, in line with those of the other vault components, and that they do so by their own—i.e., irrespective of MVP as substantiated by MVP knockdown experiments [75,79]. It is also worth mentioning that the three vtRNA isoforms clearly fulfil different roles, as documented by some examples provided above and by the observation that, unlike vtRNA1-1, vtRNA1-3 expression is increased in MDR along with the extent of its association with vault nanoparticles [4,5]. Thus, it is quite likely that much has still to be discovered regarding the involvement of vtRNAs in cellular functions.

### 3.4. Control Mechanisms of MVP Gene Expression

The control mechanisms of MVP expression are also relevant to the understanding of the physiological roles of the vault nanoparticles, as they may shed light on the scenarios whereby a cellular system requires, or not, its functions.

In one early investigation, an immunohistochemical analysis conducted on a large collection of normal and tumor human tissues revealed a diverse expression pattern, whereby the protein was broadly distributed in normal and malignant tissues [112]. With regard to normal tissues, these findings fit reasonably well with data presented in a more recent paper, showing wide expression of the MVP-encoding gene even under basal conditions, the resulting mRNAs being detected in heart, placenta, lung, liver, kidney, and pancreas [113]. Several data also evidence MVP expression in the nervous system [114,115,116,117].

In the last two decades, a broad spectrum of conditions has been identified that result in MVP upregulation. This highlights the capability of the nanocomplex of coping with stressful conditions, mostly related to exposure to anticancer drugs, as documented by an overwhelming plethora of data (discussed in Section 4) and to xenobiotics [118], but also associated with dysregulation of cellular processes, as in the case of intractable frontal lobe epilepsy [115,119,120] and synaptic plasticity [117], or with unfavorable chemical-physical conditions, as in the case of hyperosmotic stress [121]. In senescent cells, progressively increasing levels of MVP protects from apoptosis [122,123]. It is also noteworthy that MVP upregulation by LDL [124] and in breast cancer cells cocultivated with adipocytes [125], which suggests a link between lipid metabolic diseases and MVP-promoted cancer.

From 2000 onwards, insight has been provided by several investigations on the promoter sequence and the mechanisms of transcriptional regulation. The human gene contains 15 exons and is localized to chromosome 16p11.2 [80,117,126]. Its core promoter sequence includes several binding sites of transcription factors. The best known, from proximal to distal, are: STAT1, p53, a GC-box element, an E-box, a GATA-box, MyoD, an inverted CCAAT-box (termed Y-box), in addition to an upstream inducer between bases −617 and −611 for the CBF-1 transcription factor. However, no TATA core promoter element was found [127,128,129].

The upstream stimulating factor 1 (USF1), an evolutionarily conserved basic-helix-loop-helix leucine zipper transcription factor, stimulates basal activation by interacting with the E-box element, as supported by decreased MVP expression in human colon carcinoma (SW620) and human renal adenocarcinoma (ACHN) following USF1 siRNA silencing [113].

As far as the GC-box is concerned, the alignment between human and rodent sequences makes a strong conservation of GC-box element apparent, although in mice and rats three and two such elements were found, respectively, as opposed to the single one occurring in humans. These findings led to the hypothesis that this single element might also be essential for basal promoter activity. Indeed, based on several lines of in vitro and in vivo evidence, Sp1 (also mentioned in the previous Section 3.2) was shown to bind to the single GC element, thus stimulating MVP promoter basal activation [128]. Furthermore, evidence was provided that in human cells, histone deacetylase (HDAC) inhibitors (which relieve histone association with DNA, thus allowing transcription) induced strong stimulation of MVP expression, which, however, was substantially decreased by mutating the GC-box [128]. This also highlights the involvement of chromatin remodeling in MVP expression regulation [130].

A role for Sp1 was confirmed by an investigation that led to identify the aforementioned mechanism of MVP upregulation under hyperosmotic stress [121]. Under such conditions, SW620 cancer cells displayed increased Sp1 levels paralleled by its reduced ubiquitination [131]. Most notably, this study showed that, under the same conditions, specific JNK inhibitors caused decreased expressions of Sp1 and MVP. As mentioned above, JNK is a MAPK that phosphorylates several downstream proteins, thus activating the transcription of several genes and regulating the related functions, mostly prosurvival ones [100]. This signaling pathway may therefore promote Sp1-mediated MVP upregulation, consistent with the involvement of the latter in stress response. Remarkably, vault itself regulates the activation of genes under control of the c-Jun/JNK axis, as UV irradiation prevents MVP from activating the COP1 ubiquitin ligase, thus inhibiting COP1-mediated c-Jun degradation, which eventually results in transcription of the genes under control of the latter (Section 3.1) [65].

MVP expression was shown to also be under the control of IFN-γ, which in fact leads to transcriptional activation through the JAK/STAT pathway and ultimately STAT1 binding to a specific site within the MVP promoter [132]. Noteworthy, IFN-γ also stimulated MVP translation. In contrast, p53 was found to inhibit MVP expression, whereby the Y-box was identified as the relevant promoter response element (unlike the putative p53 response element). In such a case, the transcriptional repressor complex also included the Y-box-binding protein and HDAC2 [133]. A role for the Y-box-binding protein was confirmed by a paper reporting a correlation between nuclear localization of the latter and MVP expression in lung cancer specimens [134].

A further regulatory Notch1-mediated mechanism of MVP expression has been recently identified [129]. Notch1 is a transmembrane protein belonging to the family of Notch receptors. Following activation by an extracellular ligand, they are subjected to proteolytic cleavages, mostly by γ-secretase, which leads to the release of the intracellular domain (N1ICD). This is then translocated to the nucleus where it acts as a transcriptional coregulator [135]. Working on a cisplatin-resistant breast cancer cell line, Xiao and coworkers first observed that Notch1 knockdown resulted in MVP downregulation and concomitant AKT pathway suppression. Additionally, they determined that depletion of either Notch1 or MVP could reverse the epithelial to mesenchymal transition phenotype, which is typically associated with drug resistance. Furthermore, Notch1 knockdown in triple-negative breast cancer cell lines, where it is highly expressed, strongly reduced MVP expression, which was paralleled by enhanced cisplatin and DOX sensitivity. Finally, a ChIP assay showed that a Notch1 antibody could bind to a promoter region containing a putative CBF-1-binding site, located between bases −617 and −611. Actually, CBF-1 was previously identified as the essential transcription factor downstream from Notch [136]. Taken together, these results support a mechanism whereby Notch1 acts as an upstream transcriptional activator of MVP at the CBF-1 binding site, thus enhancing chemoresistance via the AKT pathway [129]. The mechanism by which vault nanoparticle activates the cascade under control of AKT is detailed in the previous paragraph (Section 3.1). The mechanisms of MVP transcriptional regulation presented above are summarized in Table 2.

In line with the picture emerging from the above data, other reports also identified mechanisms of MVP upregulation in response to the administration of several DNA-damaging agents currently employed as antitumor drugs, such as DOX and cisplatin [137,138]. In some cases (presented in Table 3), the molecular players mediating these effects have not been identified thus far. However, in a cisplatin-resistant lung cancer cell line, enhanced expression of the inflammatory factor IL-25 was detected, which resulted in NF-κB-mediated MVP level increase [139]. Nevertheless, the binding site of this transcriptional activator was not investigated. These additional findings suggest that other mechanisms of transcriptional control exist, as well as those summarized in Table 2.

In addition to the translational and transcriptional control, intracellular levels of proteins are also controlled by their degradation rates. However, in the case of MVP, very little is known in this regard. It was observed that, at least under basal conditions, MVP is subject to a low turnover, with a halving time in the order of a few days [140], suggestive of a lysosomal degradation. This implies that even a slight increase in transcription rate, as in the examples presented above, should result in major increases in protein levels. Nevertheless, evidence for ubiquitin–proteasome-sustained degradation of MVP was provided in the case of MCF7 breast cancer cells, wherein BAG3 interacted with and stabilized the vault nanoparticle, which also resulted in apoptosis resistance. In contrast, it underwent proteasomal degradation when the BAG3 gene was silenced [58]. Likewise, in pulmonary vascular smooth muscle, the proteasome inhibitor carfilzomib led to the appearance of ubiquitinated MVP [141]. This suggests that MVP may be degraded, at least in part, by the ubiquitin–proteasome pathway.

In conclusion, the scenario outlined by the available data consistently highlights the involvement of the vault nanoparticle in cell prosurvival roles and helps clarify at least some mechanisms underlying drug resistance and cancer progression.

## 4. Vault-Related Multidrug Resistance

Starting from the mid-1970s, various proteins responsible for MDR in cancer have been identified. Several mechanisms have been implicated in this response (reviewed in [152]). Most often, this was proven to rely upon the capability of some proteins to decrease drug accumulation by means of active extrusion. This is the case, in particular, of the members of the ATP-binding cassette (ABC) superfamily, which export diverse compounds in an ATP-dependent fashion [124,153,154]. The best known among these transporters is ABCB1, a transmembrane protein also known as MDR1 or Pglycoprotein (Pgp). This is widely expressed among cells types and tissues and handles a broad range of cytotoxic agents [155]. Other transmembrane glycoproteins are part of the same ABC superfamily—i.e., MRP1/ABCC1, MRP2/ABCC2, and BCRP/ABCG2 [156,157,158].

Plenty of investigations performed in more recent times reported that vault upregulation was also associated with MDR [4,144,145,159,160]. Furthermore, it was shown that U-937 human leukaemia cells selected for DOX resistance upregulated MVP and concomitantly acquired an MDR phenotype independent of other MDR proteins [143]. It was observed, however, that MVP upregulation is generally matched by a similar response by other MDR proteins [161,162,163].

The mechanisms relevant to vault-sustained MDR have been proven to be multifaceted and less straightforward than those assigned to the aforementioned drug transporters. Actually, they are only partially understood, which may be well accounted for by both the involvement of this nanoassembly in several regulatory roles and its complex molecular construction.

Starting from the very first investigations on the vault nanoparticle, it was proposed that MVP/LRP upregulation (whose mechanisms are discussed in the previous paragraph (Section 3.4)) correlates with poor prognosis and resistance to chemotherapy in cancer—e.g., ovarian carcinoma [144], acute myeloid leukemia (AML) [160], and oral squamous cell carcinoma [164]. These early findings were subsequently confirmed by plenty of data reporting MVP upregulation and drug resistance evoked by drug exposure in several cancer cell types and tissues (summarized in Table 3), although MVP upregulation may be observed in cancer even prior to drug treatment [55,112]. A recent report also suggests that forced MVP expression may be sufficient per se to induce cancer development [71]. Taken together, these findings raise the issue of what mechanisms specifically underlie vault’s capability of thwarting the cytotoxic effect of drugs. Basically, two different mechanisms have been described in a number of reports, one based on DNA-damaging agent sequestration and/or export from the nucleus, the other on uncoupling of EGF receptor (EGFR) stimulation from downstream AKT activation with resulting uncontrolled cell proliferation. Based on the available data, the latter effect is only associated with gefitinib treatment, a well-known EGFR inhibitor [82,148]. In contrast, different mechanisms participate in DNA-damaging drug sequestration/export. For instance, vault mediates DOX efflux from the nucleus and accumulation in cytoplasmic vesicles, which are then released into the extracellular medium [125] or sequestered in lysosomes [147] (Table 3). A similar vesicle-mediated efflux mechanism has been proposed for bleomycin, although possible drug handover from vault to drug efflux pumps has also been suggested [98,162]. Likewise, vault has been implicated in similar mechanisms of cisplatin efflux. However, in this case, evidence was provided suggesting that vtRNA(s) may also be involved in vault-supported drug clearance, as documented by increased cisplatin sensitivity in cancer cells with reduced vtRNA expressions via RNA polymerase inhibition [149]. In line with this report, direct binding of mitoxantrone to well-defined regions of vtRNA1-1 and vtRNA1-2 was demonstrated, as well as enhanced drug resistance in several vtRNA-overexpressing cancer cell lines [150,151]. This adds to other gene regulation effects ascribed to vtRNAs—i.e., GAGE6 drug resistance gene silencing [79] and probable activation of CYP3A4 gene, which codes for an isoform of cytochrome P450, an enzyme involved in drug detoxification [77] (Section 3.1 and Table 1).

Whereas MVP stands out as the key player in phenomena related to MDR, knowledge regarding the roles of the minor vault proteins is sparse. An in-depth understanding of how these components contribute to the physiological roles of the vault nanocomplex is complicated even more by the fact that they are predominantly not associated with the nanoparticle. As mentioned in Section 2, TEP1 is also a noncatalytic subunit of the mammalian telomerase complex, whereas vPARP is involved in DNA repair and metabolism, and also localizes to the nucleus and the mitotic spindle, suggestive of multiple functions being supported by this protein [6]. 

Different reports highlight coordinated expression of the three vault proteins, as in the case of a drug-resistant SCLC cell line, wherein MVP overexpression also increased vPARP and TEP1 levels [165]. Likewise, a recent work also provides evidence of coordinated expression, in that tunicamycin treatment of a DOX-resistant LoVo human colon adenocarcinoma cell line completely suppressed the expression of all three, although the mechanism underlying this phenomenon was not elucidated. The same investigation also showed that in the resistant ovarian carcinoma A2780TR cell line, MVP knockdown resulted in vPARP downregulation and vice versa [166]. Overall, such expression mechanisms substantiate the hypothesis that the three proteins act coordinately, at least to some extent, to fulfil their physiological roles. Nevertheless, the minor vault proteins are also apparently dispensable just as MVP is, as both vPARP- and TEP1-deficient mice were viable and fertile, with no obvious phenotypic changes induced by the deletion [37,38]. Remarkably, evidence supporting a direct association of vPARP and TEP1 was also provided in HEK293T cells that do not express MVP, suggestive of a functional cooperation even in a non-vault-associated form [38].

Regarding a possible role for vPARP and TEP1 in cell proliferation and drug resistance, the picture emerging from the available literature mostly confirms their prosurvival role and coordinated expression mechanisms. An investigation conducted on postsurgery ovarian cancer samples from patients not treated with anticancer drugs detected reduced levels of mRNAs encoding all three vault proteins relative to healthy individuals but, surprisingly, this was paralleled by higher protein expression, which points to post-translational regulation mechanisms [163]. In line with these findings, in drug-resistant ovarian cancer cell lines, both MVP and vPARP knockdowns resulted in decreased cell viability [166].

Despite the large amount of investigations presented above, there are still different views, due to conflicting results, as to whether and how vaults are involved in MDR. Nevertheless, there is no doubt that, at least in some cases, vaults directly support drug resistance mechanisms (Table 3). For instance, the mechanisms by which they prevent the antiproliferative effect of gefatinimb [82,148] and the DNA-damaging effect of mitoxantrone [150,151] are well-elucidated. Yet, their roles are still a matter of debate, due to apparent inconsistencies between investigations that detected association between MVP upregulation and MDR and others that failed to do so. For instance, no MVP upregulation was detected in autopsy samples of lung cancer cells exposed ante-mortem to platinum [167]; conversely, embryonic stem cells and bone marrow cells of MVP^−/−^ mouse models did not display hypersensitivity to various cytostatics [168]; likewise, MVP knockdown in non-SCLC cell lines did not affect survival of DOX-treated cells [169]. A recent paper even reports positive correlation between high bone marrow LRP/MVP expression and a favorable therapeutic outcome [170]. Furthermore, at least in the mentioned case of MVP^−/−^ mouse models, no compensatory upregulation of other MDR proteins was detected, which is a likely response for the preservation of drug resistance [168]. Although a possible role for the minor vault proteins might be invoked as vPARP^−/−^ mice were proven to be more susceptible to carcinogenic-induced colon and lung tumors [40], this is not likely either, as vPARP and TEP1 levels are under the control of MVP, as mentioned above. Although this issue is still open, one might speculate that other, as yet unidentified drug resistance-related proteins exist. Furthermore, as mentioned above, MVP is subject to PTMs such as phosphorylations and S-glutathionylation, which might involve possible changes in its functional state and result in diverse responses, also depending on the cellular milieu.

**Figure 2:**
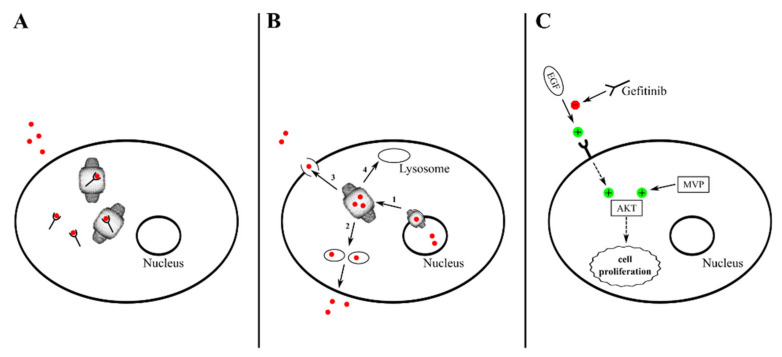
Mechanisms of vault-sustained drug resistance. (**A**) Some DNA-damaging drugs may be sequestered by vault-associated RNA molecules (vtRNAs). (**B**) DNA-damaging drugs may also: (1) be exported from the nucleus; (2), accumulate in cytoplasmic vesicles and undergo exocytosis; (3) be handed over to drug efflux pumps; (4) be sequestered in lysosomes. (**C**) MVP uncouples, by an unknown mechanism, protein kinase c (AKT) activation from EGF receptor (EGFR) stimulation, thus thwarting the inhibitory effect of gefitinib on the EGF/AKT signaling axis.

In Figure 2, some major MDR mechanisms are outlined.

Based on the large body of research presented above, MVP upregulation has also been identified as a prognostic biomarker for radio- and chemotherapy resistance and as having poor clinical outcomes in plenty of cancer diseases. This holds true, in particular, for several carcinomas—for instance, HCC [171], non-SCLC [134], ovarian [144], oropharyngeal [172], oral squamous cell [164], and cervical [173] carcinomas. Likewise, in the case of blood and immune cell cancers, such as AML [160], multiple myeloma [174], and adult T-cell leukemia [175], MVP has also been proven to be prognostic of poor outcome. In some cases, the predictive ability is improved by combining high upregulation data of other proteins with that of MVP. This is the case, for instance, of HCC and cervical carcinomas, whereby MVP data were combined with those of betaine-homocysteine S-methyltransferase and Bcl-2/IGF-1, respectively.

The prognostic potential of MVP in diseases other than cancer, such as epilepsy, is also noteworthy, as shown in a study reporting MVP and BCRP upregulation in surgical resections from drug-resistant epilepsy patients, which was suggested to be predictive of pathology-specific phenotypes [120]. Still in the context of neurological diseases, the role of vtRNA2-1 is also remarkable, which is also in part associated with the vault nanoparticle [53]. Actually, upregulation of a small vtRNA2-1-derived fragment was recognized as a common trait in brain areas suffering from Parkinson disease [52,176]. In turn, elevated levels of this fragment likely result from increased expression of vtRNA2-1 itself, which occurs early during the progression of this disease and has therefore been suggested as a diagnostic biomarker. Finally, serum MVP anti-MVP autoantibodies have also been proposed as biomarkers in rheumatoid arthritis [177].

In conclusion, although many of the approaches detailed above may require further refinements, they suggest, nevertheless, that the development of prognostic and diagnostic tests based on the detection of MVP, and possibly of the other vault components, represents a promising field of investigation.

## 5. Investigations on Vault Localization and Trafficking Provide Further Hints on Its Biological Roles

Plenty of investigations on the vault nanoparticle have also focused on its intracellular localization and trafficking mechanisms, which has uniquestionably helped better understand its biological roles. Provided that a predominantly (>90%) cytoplasmic vault localization is well-established [9], its involvement in nuclear import/export was first suggested in a paper reporting its localization at the nuclear pore complex (NPC), also remarking that its mass, diameter, and radial symmetry make it suitable for this interaction, suggestive of vaults docking at the NPC [178]. This was confirmed by a more recent study carried out on cortical neurons, whereby immunolocalization experiments demonstrated vault/NPC association [116]. These findings are suggestive of an involvement of vaults in nucleocytoplasmic transport. However, some investigations performed on different biological specimens could only detect localization to the perinuclear region [179,180]. In contrast, intranuclear localization was confirmed by several other reports ([181] and the literature therein), in keeping with some observations implicating vault in protein nuclear import [54,55,58] (Section 3.1 and Table 1). Particularly, it was enriched around the nucleoli, which might be related to its tendency to copurify with ribosomes, although the physiological significance of such an interaction has not been elucidated [182,183].

Regarding the mode by which vaults participate in nuclear trafficking, there are apparently conflicting results. For instance, PTEN binds to MVP via its putative NLSs and is then imported into the nucleus, as outlined in Section 3.1. Thus, MVP would act in the process as an importin [83,84,85]. In contrast, another experimentation identified MVP as a cargo protein of the importin subunit KPNA7 [184]. Whatever the mechanisms may be, current knowledge substantiates the hypothesis that vault can shuttle between nucleoplasm and cytoplasm. Interestingly, in sea urchin, a substantial relocalization of MVP from the cytoplasm to the nucleus was detected during embryogenesis [183], which suggests that the process is under control of some as yet unidentified effector. Vault shuttling between nucleus and cytoplasm would not only support protein exchange but also drug extrusion from the nucleus and the resulting MDR (Section 4 and Table 3).

Vault cytoplasmic dynamics were also investigated in non-SCLC [180] and PC12 cells [185] using the nanoparticle in fusion with the green fluorescent protein (GFP). In either cellular context, a small fraction was found to be associated with intact microtubules, which allowed fast and directional migration, probably enabling cargo protein transport towards the nucleus. Microtubule-bound or unbound vaults would conceivably also migrate towards the plasma membrane, which would eventually result in release into the extracellular medium of drugs compartmentalized in their hollow cavities. A study performed on human bladder cancer cells showed DOX accumulation in lysosomes, which was abolished by MVP knockdown. Silencing was also paralleled by a major disruption of the lysosomal compartment, as shown by altered staining of some lysosomal markers [147]. It was concluded that MVP plays some important role in preserving lysosomal function. Furthermore, in the different cellular context of breast cancer cells cocultured with adipocytes, vaults accumulated in cytoplasmic vesicles, which were then expelled from the cells [125]. Although the details of this vesicular trafficking have to be elucidated, these two reports substantiate the hypothesis that extracellular vesicle secretion may represent a pathway of vault-mediated drug disposal. It has been suggested, in addition, that the process might also be accomplished by drug handover from the nanoparticle to the ABC transporters [98,162].

The intracellular trafficking of vaults is apparently a complex process, as suggested by other investigations. Indeed, the nanocomplex was found in different locations in addition to the above-mentioned ones—e.g., in the neuritic tips of PC12 cells, colocalized with actin filaments [114], and in lipid rafts, either in macrophages, in association with SR-A, as discussed in Section 3.1 [68], or in human lung epithelial cells infected with *Pseudomonas aeruginosa*, where it plays a role in bacterial clearance [186]. 

Interestingly, vaults were also shown to undergo reversible polymerization at low temperature (21 °C), thus giving rise to the so-called vault tubes, in a process that might be promoted by the concurrent microtubule destabilization [31]. However, the physiological relevance of this phenomenon has still to be defined.

Another recent paper reports on MVP being localized to the surface (referred to as csMVP) of various HCC cell lines and other cancer cells but not of normal hepatocytes [187]. This phenotype was associated with much higher clonogenic cell survival. It was also found that both ERK and mTOR pathway inhibitors significantly decreased cell surface expression of MVP, suggesting that its translocation is regulated by ERK and mTOR signaling. In turn, antibodies directed to csMVP inhibited ERK and mTOR signaling and, likewise, MVP knockdown substantially decreased cell proliferation. Instead, csMVP was increased under serum starvation used as a representative stressful condition. Overall, these results suggest a role for csMVP in cancer progression. As the trafficking mechanisms sorting the nanocomplex to cell surface have not been investigated, it is not known, so far, whether csMVP localizes to lipid rafts.

Still regarding vault trafficking, the finding that advanced mouse colon cancer cells secrete exosomes containing MVP in association with miR193a, a tumor suppressor miRNA [188], is also noteworthy. Exosomes are vesicles ca. 100 nm in diameter, which arise from multivesicular bodies through exocytosis and, akin to other extracellular vesicles, can deliver molecular signals to other cells [189]. MVP-mediated miRNA193a extrusion resulted in active cancer cell proliferation, whereas MVP knockout was paralleled by intracellular miR193a buildup with ensuing inhibition of tumor progression [188]. Thus, these findings reveal another mechanism by which vaults sustain tumor progression and, in addition, are suggestive of extracellular vault occurrence, also in keeping with the following pieces of evidence: (1) they are nonimmunogenic [190]; (2) they undergo clathrin-mediated endocytosis [191]; (3) MVP is immunologically detectable in serum [69,70].

Taken together, these results open the largely unexplored scenario of vault extracellular trafficking, which might possibly support intercellular cross-talk mechanisms. 

## 6. The Evolutionary History of the Vault Nanoparticle

From an evolutionary perspective, the vault nanoparticle displays baffling properties. Starting from the earliest studies, it was shown that it is widespread among eukaryotes, yet no MVP-encoding genes were found in fungi, insects, nematodes, and possibly in plants [18,45]. Even more surprisingly, despite the large intracellular amounts of MVP, it was shown that MVP knockout mice are viable, healthy, and show no obvious abnormalities [167,192], although it should also be pointed out that some reports also highlight its capability to effectively cope with different cell stress conditions [61,69,72,73,122].

Taking into account the peculiar properties of this nanocomplex, a bioinformatic analysis should be the most effective approach to reconstruct its evolutionary history, which might possibly also help infer the origin of its diverse biological roles. Most of bioinformatic data available to date have been reported in two papers by Daly and coworkers [19,193]. The main bioinformatic tools they employed were: BLAST for the search for related sequences; I-TASSER [194] for the prediction of tertiary structures; RosettaDock to assess possible side-by-side assembly of the MVP monomers [195]. The main control structure used was the rat MVP sequence, also constrained by the crystal structure [12]. 

Initially, matching sequences were found in kinetoplasts of excavates, oomycetes (stramenopiles), and paramecium (an alveolate). Using a more permissive threshold, two MVP putative sequences were identified in the excavate *Naegleria gruberi* (belonging to the clade Heterolobosea). Based on I-TASSER and RosettaDock computing analyses, the authors drew the likely conclusion that both of them are capable of forming a vault complex, despite being the most divergent from the rat counterpart among excavated MVPs, with sequence homologies of less than 20% [193]. A strong divergence in sequence may be compatible with the retention of the tertiary and quaternary structures, given that only residues essential to maintain the shape and lateral docking between adjacent polypeptide chains must be conserved.

The significance of this finding relies on the fact that the *N. gruberi* genome is believed to retain features close to the early stages of eukaryotic evolution [196]. This substantiates the hypothesis of an *mvp* gene already occurring in the last eukaryotic common ancestor (LECA), which in turn implies that in some eukaryotes it was either lost or evolved beyond recognition.

In a more extended study, the occurrence of MVP in the major groups of eukaryotes was investigated [19]. By a simple BLAST analysis, several matching sequences were found among metazoan, amoebozoa, and kinetoplasts of excavates. They were instead less represented in stramenopiles (the only identified sequences coming from two ciliate species), whereby I-TASSER criteria were required for their identification. Among opisthokontes (that include all metazoan and fungi) notable absences were observed in arthropods and fungi. Regarding possible occurrence of MVP in plants, conflicting results were obtained. Initially, a rice and a barley variant were found, the latter displaying a 55% homology with rat. However, based on their data, the authors judge that this is more likely to be a contaminating sequence from amoebozoa. So, no clear-cut evidence supporting the existence of plant MVP varieties is so far available. On the whole, these studies have highlighted MVP occurrence even in the LECA and helped reconstruct the relevant sequence. Thus, these conclusions might be validated by cloning and expressing the putative ancestral MVP gene and analyzing structural and biological properties of the resultant vault.

Concerning the possibility that MVP ancestors may have arisen in prokaryotes, the available data do not currently allow us to draw unequivocal conclusions. Actually, the analysis may be flawed by horizontal gene transfer despite a number of putative homologs found in bacteria [19]. Nevertheless, BLAST analysis identified two proteins—i.e., TolA and band 7 protein, ubiquitous in prokaryotes, whose sequences fall within default identification parameters. Although the significance of this finding deserves further investigation, it possibly points to the origin of MVP being rooted in prokaryotes.

This bioinformatic analysis, while substantiating the idea that vault nanoparticles were already present in LECA, also highlights the issue of its primordial functional role and evolution. Quite likely, a hollow assembly that big should possess a natural propensity to encapsulate substances of different types. In line with this assumption is its capability to open at low pH [197], thus allowing the uptake of molecules, preferably anionic, driven by the interaction with the positively charged interior [198]. The well-known involvement of vaults in detoxification processes might be related to this capability [199], which also might play a protective role from harmful bacteria, in case they were engulfed by eukaryotes. Vault loss in plants and fungi might be accounted for by the fact that they do not generally consume bacteria. Likewise, its loss in insects might be explained by their hosting of complex symbiontic, bacteria-containing communities, which protect them from plant pathogens and toxic chemicals [200].

As far as the vault-associated molecules is concerned (i.e., vPARP, TEP1 and vtRNAs), they obviously underwent an (at least in part) independent evolutionary process, as all of them also occur in isolation. Nevertheless, the repertoire of the molecules included in the nanoparticle quite probably changed during evolution, in keeping with their diversity in the extant variants. For instance, whereas the several PARP variants are subdivided into six clades, the extant vPARP belongs to clade 5 and is only found in metazoa and amoebozoa, suggestive of a recent adaptation [25]. In any case, the issue of how MVP and vault-associated molecules may have coevolved still deserves in-depth investigations.

Another interesting hypothesis regarding vaults’ evolutionary origin maintains that it might be a relic of an early viral symbiont of the eukaryotic cell [53]. This view is supported by the following pieces of evidence: they are very large assemblies composed of multiple copies of a single polypeptide chain enclosing a large central cavity; they can self-assemble in insect cells, just as viruses do [14]; the promoter arrangement of some viral RNAs is also shared by vtRNAs, in that they are dependent on both internal and external promoter elements. This hypothesis, however, can hardly be reconciled with the well-documented analysis performed by Daly and coworkers [19,193].

## 7. Vault as a Tool for Drug Delivery

Thanks to their unique properties, vault nanoparticles represent ideal nanovectors as they feature several properties that are desirable as therapeutic delivery systems. In particular, in addition to the huge internal cavity, their size prevents them from being taken up by kidney or liver; they can freely access the draining lymph nodes when injected intradermally; they also offer some degree of protection to the hosted proteins from external proteases; they are biodegradable; finally, and no less important, they are nonimmunogenic, as substantiated by several pieces of evidence [190,201].

When designing a nanoparticle for therapeutic, diagnostic, and imaging applications, most efforts are devoted to fulfilling two basic requirements: (i) it must be capable of selectively targeting the desired cell line(s); (ii) it must effectively carry suitable molecules endowed with the desired properties (e.g., cytotoxicity, therapeutic potential, etc.). In the case of the vault nanoparticle, these goals can be achieved by taking advantage of both genetic and chemical tools.

Notable results have been attained by genetically engineering the MVP-encoding gene. As the C-terminus of MVP is localized at the caps and exposed on the surface of the nanoparticle, whereas the N-terminus is close to the waist, protruding into the interior, an ideal approach to selectively target this nanoparticle can rely upon genetic fusion of a suitable peptide at the C-terminus. Actually, unfunctionalized vaults are taken up neither specifically nor efficiently by HeLa [11,202] and glioblastoma cell lines [191]. In the latter case, it was also shown that the process is sustained by clathrin- but not caveolae-mediated endocytosis.

Effective vault genetic engineering was achieved by adding a C-terminal peptide extension to MVP [11] using an engineered 33-amino-acid-long fragment, of protein A’s Z domain that retained high affinity for the Fc-binding peptide of IgG [203]. This vault variant lends itself to be bound to any IgG, which represents a general tool to target the nanoparticle to specific surface antigens. In the case described, it was bound to a monoclonal anti-EGFR antibody. The conjugated vault specifically targeted A431 carcinoma cells, where the receptor is overexpressed as in many other cancer cell lines. Likewise, strong binding capacity to the same target was also attained by fusing EGF itself to vault C-terminus [11].

To package foreign proteins into vaults, an effective approach takes advantage of the INT-binding site of MVP, located at residues 113–221, approximately corresponding to the third and fourth repeat domains [204]. A shortened INT variant, 147 residues long, was used for this purpose and as a proof of concept it was first shown that luciferase-INT did bind to vault [197,202]. Notably, it was also shown that the packaging did not require cotranslation but could take place by just mixing the two components [30]. Following these initial findings, many other proteins were packaged into the vaults. Worthy of mention are, in particular, pVI, CCL21, and major outer membrane protein (MOMP) [190].

pVI is an adenoviral protein whose N-terminal region includes a peptide (residues 34–54) with predicted amphipathic helical structure, endowed with membrane lytic activity. This should allow endosomal escape and improve penetration into target cells [205]. Thus, vaults were either packaged with the pVI lytic peptide in fusion with INT (pVI-INT), or produced with this same peptide directly in fusion to MVP N-terminus [10,206]. In either case, the nanoparticle could effect endosomal escape, pVI in fusion with MVP being more effective in promoting the release into the cytoplasm. When the construct was also fused with the Z domain at the C-terminus (pVI-MVP-Z) and bound to anti-EGFR antibodies, an even more efficient lytic vault was obtained. Both the ribotoxin saporin and a GFP-encoding plasmid underwent facilitated uptake by a macrophage cell line when preincubated with vault packaged with pVI-INT, but not with INT alone [206].

In this context, it is also worth mentioning another modification of the N-terminus, which was also performed genetically and consisted of the addition of a 12-amino-acid sequence (referred to as CP) containing four cysteine residues. This resulted in much greater nanoparticle regularity and stability, probably due to disulfide formation between adjacent MVPs [42,207]. 

Scanty water solubility and the ensuing poor bioavailability are major constraints in hydrophobic drug delivery. To address this issue, two different types of vault-based nanodevices were developed. In one case, a truncated form of apolipoprotein-AI consisting of a number of amphipathic helices fused with the INT domain was used. This construct spontaneously incorporated a mix of phospholipids and the hydrophobic drug all-*trans* retinoic acid, thus giving rise to hydrophobic nanodisks. They were stably packaged into CP-MVP vaults by just mixing these two components, which also offered protection against drug loss [208]. All-*trans* retinoic acid is a therapeutic agent effective in the treatment of different diseases, including cancer, although it is also endowed with significant toxicity [209]. Thus, its packaging might also help prevent, in principle, its delivery to off-targets. Conversely, this nanodevice exerted enhanced toxicity against the HCC cell line HepG2 compared with free all-*trans* retinoic acid, which represents a promising achievement towards the development of an innovative platform for the delivery of water-insoluble drugs.

Another similar approach took advantage of the N-terminal 31-residue fragment of the NS5A protein from the hepatitis C virus, which was fused with the MVP N-terminus. The peptides folded into amphipathic helices with an asymmetrical distribution of charged residues, which associated with each other via ionic and van der Waals interactions, thus forming a lipophilic ring within the vault lumen and close to the waist [210]. Once stripped by treatment with a nonionic detergent of the lipophilic molecular components incorporated during expression in insect cells, vaults were proven capable of selectively binding apolar drugs, as opposed to hydrophilic ones. Specifically, in addition to the aforementioned all-*trans* retinoic acid, Amphotericin B (an antifungal antibiotic) and Bryostatin 1 (a multipurpose therapeutic lead) were also effectively bound by preincubation with the nanoparticle. It was estimated that up to thousands of hydrophobic molecules could bind to each vault. Thus, this approach provides a platform for future development of nanodevices capable of targeted drug delivery. 

Other promising approaches of therapeutic relevance also took advantage of the capability of MVP to bind proteins via the INT domain. In particular, the major outer membrane protein (MOMP) and the PmpG protein from *Chlamydia muridarum* (highly immunogenic membrane proteins) were produced as fusion constructs, (MOMP-INT and PmpG-INT, respectively). CP-MVP-Z vault constructs packaged with either constructs were successfully used for intranasal vaccination in a murine *C. muridarum* genital infection model [211,212]. Likewise, when engineered vaults packaged with CCL21-INT (CCL21 being a chemokine able to attract a variety of immune cells) were delivered intratumorally into murine tumor models—i.e., either Lewis Lung tumor [213] or glioma [214]; this resulted in a substantial growth inhibition. It should be pointed out that the effectiveness of these treatments quite likely relies, at least in part, upon the slow release of the therapeutic agents from the nanoparticle. Based on a similar strategy, a further nanodevice destined for tumor therapy was more recently developed by packaging into the vault the NY-ESO-1 peptide in fusion with the INT domain. When incubated in the presence of CP-MVP-NY-ESO-1-INT vaults, native dendritic cells underwent maturation, thus inducing antitumor response [215]. These results await further developments for moving from the preclinical to the clinical stage.

As outlined above, many experimental designs aimed at developing new therapies rely upon the remarkable capability of the vault nanoparticle to open and close, thus exchanging molecules between the interior and exterior, a property referred to as “breathing” [30]. Indeed, this justifies its capability of spontaneously incorporating, even in the test tube, vPARP via the INT domain and TEP1, as well as slowly releasing therapeutic agents and exposing the pVI-INT peptide, which mediates endosomal escape [206]. A subsequent contribution provided further insight into this phenomenon by showing that, in vivo, individual vault nanoparticles can exchange MVP subunits with each other, a process probably supported by rapid separation and reassembly at the waist, suggestive of a half vault exchange mechanism [17]. In addition to the dynamic nature of the nanocomplex, another factor affecting the exchange of INT-containing proteins between the interior and exterior should be INT affinity for its binding site. Based on this concept, two MVP variants were produced, which were endowed with lower and higher affinities, respectively, as compared with that of the wild type (262 nM) [24]. Such fine tuning should make it possible, in principle, to modulate the molecular release of cargo proteins depending on the experimental and therapeutic requirements. In this context, it is also worth noting that vaults were shown to disassemble at pH 3.4, each half giving rise to a flowerlike structure [216], although it seems likely that these conformational changes already start at pH > 4 [197,217]. This mechanism might favor the release of encapsulated molecules into the acidic milieu of endosomes and lysosomes, but it is unlikely that it will be exploited for molecule packaging into vaults, due to its irreversibility [197].

Interestingly, when a cell-penetrating peptide, namely the basic fragment, 13 residues long, of TAT protein from HIV1 (GRKKRRQRRRAHQ) was fused at MVP C-terminus, this strongly boosted vault binding and internalization by a variety of cell types [218]. As TAT-mediated uptake is a nonspecific one, it is expected that such vault modification might be helpful in potentiating the antitumor effects of CCL21-vaults when directly injected into tumors [190].

Complementary to genetic engineering are chemistry-based modifications, which can also confer new properties of biotechnologyical/biomedical relevance on nanoparticles. For instance, CP-MVP vaults were derivatized with modified poly(N-isopropylacrylamide) chains carrying α-dansyl fluorophores at the free ends and capable of forming disulphides with free cysteines. This polymer undergoes a temperature-dependent, reversible phase transition, whereby it becomes water-insoluble above its lower critical solution temperature. Thus, when heated, the nanoparticles underwent reversible aggregation in the temperature range of about 35–45 °C as documented by different analytical methods. It is suggested that these devices might be useful for sustained drug release in tumor cells when combined with in vivo localized heating techniques [219]. As a refinement of the same approach, the vault was conjugated with a modified form of the above polymer, i.e., poly(*N*-isopropylacrylamide-*co*-acrylic acid), whose lower critical solution temperature was pH-dependent, whereby the transitions were detected at 31.9 °C at pH 5, 44.0 °C at pH 6 and above 60 °C at pH 7. It was suggested that this technology might allow selective drug delivery to some solid tumors by taking advantage of their acidic environments [220].

**Figure 3:**
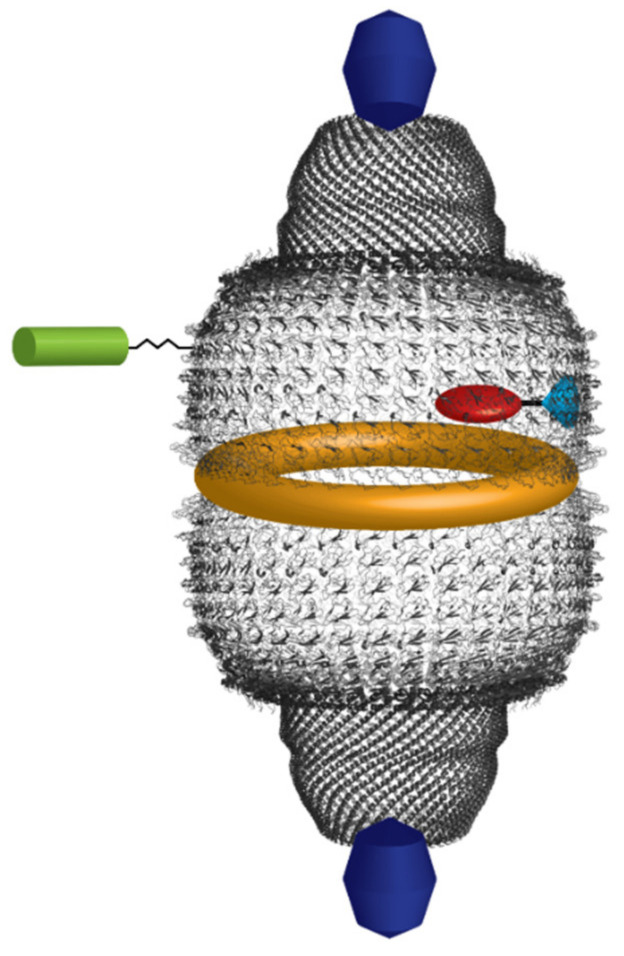
Some basic vault engineering strategies. Orange: the 12-amino-acid (CP) peptide in fusion at the MVP N-terminus confers much greater nanoparticle regularity and stability. Purple: proteins genetically fused at the N-terminus of the INT domain (green) are packaged into the vaults for facilitated delivery (e.g., the pVI endosomolytic peptide, the CCL21 chemokine, the immunogenic major outer membrane protein (MOMP), and PmpG proteins, fluorescent proteins, the NY-ESO-1 peptide, which stimulates the maturation of dendritic cells). Blue: peptides in fusion with MVP C-terminus are exposed on the surface of the nanoparticle and can either target it to specific cell receptors (e.g., EGF, the Z domain of protein A, which can bind any IgG) or facilitate its cellular uptake (e.g., pVI and the cell-penetrating peptide TAT48). Red: different proteins, such as TAT48 or fluorescent probes, can be covalently attached to MVP subunits via suitable linkers.

A more recent contribution further expanded the scope of vault conjugation approaches and the ensuing application potential by implementing a wide repertoire of chemical modifications [207]. In particular, the investigation took advantage of the huge number of lysine side chain amino groups and cysteine thiols, some of which were derivatized with different fluorophores using suitable synthetic procedures. The cell-penetrating TAT48 peptide was also conjugated to the nanoparticle, which resulted in higher cellular uptake compared with unmodified vault. Overall, this work made it possible to doubly modify vaults with both cell-penetrating peptides and imaging agents, thus developing nanodevices suitable for research and clinical applications, such as imaging, targeted delivery, and enhanced cellular uptake. Although these chemical approaches may be less specific compared with genetic ones, they are less time consuming and, as a matter of fact, have disclosed a much wider diversity of vault modifications. Figure 3 summarizes some engineering approaches described above.

## 8. Concluding Remarks

In the last two decades, the knowledge on the roles of the vault nanoparticle has greatly expanded, revealing an astoundingly complex picture of its involvement in cellular processes. Although many key issues are still unanswered, an initial understanding of its biological role(s) and significance is emerging. Mostly, this macromolecular complex fulfils roles aimed at preserving cell viability by coping with cellular stresses, including detoxification, protection from infections, DNA-damaging agents, irradiation, hypoxia, hyperosmotic, and oxidative stresses. Unfortunately, some of these capabilities also result in MDR. These diverse cellular functions are accomplished by different mechanisms, mainly gene expression reprogramming under MVP control, activation of proliferative/prosurvival signaling pathways, export from the nucleus of DNA-damaging drugs, and import of specific proteins.

Whereas the most recent advancements have made substantial contributions on several aspects of vault’s involvement in cellular processes, they have also raised a number of new questions that need to be addressed. For instance, the most recent achievements have led to the identification of no less than fifteen different proteins interacting with MVP (Table 1), mostly key players in signaling pathways. This prompts a question of considerable importance, namely whether and how such pathways may interact with each other via MVP binding. Relevant to this issue is the identification of the interacting regions of MVP, which so far was achieved only in few cases. Specifically, PTEN and PERK bind to putative NLS sequences and to the middle domain, respectively (Section 3.1). This issue is worthy of further exploration, as it might make it possible to identify mutually exclusive (or cooperative) interactions.

In addition to pathway-controlling mechanisms based on noncovalent interactions, vault PTMs represent a further regulatory layer that quite likely plays key roles in modulating its functions. In this respect, MVP phosphorylation in the cellular milieu was first observed in stomach cancer cells and was shown to be effected by Src protein kinase, playing a role in ERK pathway regulation [62]. More recently, MVP S-glutathionylation in airway smooth muscle cells was also discovered and shown to trigger an antiapoptotic response, probably under control of the redox status [64]. These findings seem to be only the tip of the iceberg, so PTMs will likely represent an active field of future research.

Another puzzling issue regards how different vault components may complement each other in their functions. Very little is known in this respect, and the issue is even more complicated by the fact that the minor vault proteins and vtRNAs are for the large part non-vault-associated. However, their functional roles are to some extent related in that they generally share protective and prosurvival capabilities. Furthermore, some observations provide circumstantial evidence that, at least to some extent, their actions are coordinated. This is suggested, for instance, by vPARP and TEP1 expressions being controlled by MVP levels [165], or direct associations of vPARP and TEP1 in cells that do not express MVP [38].

As a further surprising feature, vaults are apparently dispensable for survival, at least under most biological scenarios, despite the remarkable investment made to synthesize up to 10^4^–10^5^ particles per cell [20]. Although this issue deserves further investigations, it should be pointed out, nevertheless, that their requirement becomes apparent under specific stressful conditions. It protects, for instance, against obesity and atherosclerosis [73], viral infections [69,72], and apoptosis in senescent fibroblasts [122]; it also promotes Elk1-mediated and EGF-dependent transcriptional activation, thus supporting cell survival [61]; likewise, it prevents growth inhibition under conditions of nutritional stress in *Dictyostelium* amoebae [221]. Given the diversity of mechanisms whereby the vault nanoparticle fulfils such protective and prosurvival roles, it is conceivable that only recently, on an evolutionary time scale, it undertook these roles.

In conclusion, although some key issues regarding the biological roles of the vault nanoparticle still require a better understanding, future research promises to deliver useful hints to develop novel strategies for the treatment of diverse diseases, most notably cancer. From a clinical perspective, a project aimed at exploiting vaults packaged with CCL21-INT for lung cancer therapy is currently in phase I [213], whereas MVP immunohistochemistry makes is possible to reliably identify potential markers of malignancy in uterine smooth muscle tumors [222]. Thus, it is to be expected that in the near future the vault nanoparticle will find further and broader applications as both a therapeutic and a diagnostic tool.

## Figures and Tables

**Table 1:** The involvement of the vault nanoparticle in signaling pathways.

Cellular Pathways and Mechanisms under Control	Vault Components	Interactor(s)	Tissue/Cells	Cellular Effects	Reference
Estrogen signaling	MVP	Estrogen receptor	MCF-7 breast cancer cells	Vault translocation to the nucleus	[54]
EGF/PI3K/AKT	MVP	PTEN	Glioblasotoma	PTEN nuclear import	[55,56]
ER stress	Probably MVP	PERK	Nonsmall cell lung cancer (H1299)	Vault retention in cytosol	[57]
BAG3	MVP	BAG3	Several breast cancer cell lines	Vault nuclear import	[58]
YPEL4	MVP	YPEL4	COS-7 fibroblast-like cells	Reduced Elk-1 activation	[59]
ERK	MVP	B7-H3	Human mammary epithelial cells	MEK activation	[60]
ERK	MVP	SHP-2 (*)	Human embryonic kidney 293 cells; mouse embryo fibroblasts	Elk-1 activation; prosurvival	[61]
ERK	MVP	Src (**)	stomach	Possibly prosurvival	[62]
14-3-3ε	MVP	14-3-3ε	Hepatocellular carcinoma cells	MVP-induced DR inhibition	[63]
IL-22/PDGF/STAT3/AKT	MVP	Myosin-9	Smooth muscle cells	STAT3 and AKT activation; apoptosis inhibition	[64]
c-Jun	MVP; probably also vPARP	COP1 (E3)	Human embryonic kidney 293 cells; HeLa cells	c-Jun-mediated response to UV stress	[65]
Hypoxia signaling	MVP	HIF-1α	Human renal adenocarcinoma ACHN cells (ACHN)	Favoring hypoxia adaptation	[66]
SR-A receptor	MVP	SR-A	Mouse peritoneal macrophages	TNF-α production; apoptosis	[67]
Apoptotic pathway	MVP	Caspase-1; caspase-9 (***)	human primary keratinocytes; human primary fibroblasts	Antiapoptotic effect	[68]
Innate immune response to HCV	MVP	unknown	Peripheral blood mononuclear cells; Huh7 hepatoma cells	MVP upregulation; type-I IFN activation	[69]
Innate immune response to HBV	MVP	MyD88	Peripheral blood mononuclear cells; HepG2 and HuH7 hepatoma cells	NF-κB and IFN-β activation	[70]
p53	MVP	IRF2	Mouse liver	p53 degradation	[71]
Proinflammatory response to influenza A virus	MVP	c-Fos; C/ERBβ-LAP; p50/p65	epithelial A549 cells; peripheral blood mononuclear cells	Virus-evokedIL-6 and IL-8 production	[72]
Modulation of inflammation; prevention of metabolic disorders and atherosclerosis	MVP	TRAF6	Macrophages	Regulation of NF-κB-dependent transcriptional effects	[73]
Unknown	vtRNA	La autoantigen	HeLa cells	Unknown	[8]
Autophagy	vtRNA1-1	sequestome-1/p62	Several human and murine cell lines	Antiautophagic effect	[74]
EBV infection	vtRNA1-1	unknown	Burkitt lymphoma BL2 cells	Antiapoptotic effect	[75]
PI3K/AKT and ERK pathways	vtRNA1-1	unknown	Hela cells	Antiapoptotic effect	[76]
Gene expression	vtRNA1-1(via svRNAb)	Dicer and Argonaute proteins	MCF-7 breast cancer cells	CYP3A4 (P450-expressing) gene silencing	[77]
Gene expression	vtRNA1-1(via svRNA4)	NSUN2 methylase; SRSF2	Human dermal fibroblasts	Inhibition of keratinocyte differentiation	[78]
Gene expression	vtRNA1-1	PSF	MCF-7 breast cancer cells	GAGE6-MDR gene activation	[79]
Gene expression	vtRNA	Unknown	*Trypanosoma brucei*	*Trans*-splicing	[45]

* MVP dephosphorylation; ** MVP phosphorylation; *** MVP proteolytic cleavage.

**Table 2:** Binding sites of transcription factors (from proximal to distal) of human MVP promoter and their transcriptional effects (when known).

Binding Site	Transcription Factor	Transcriptional Effect	Reference
STAT1	STAT1	Activation	[132]
p53	-	-	[127]
GC	Sp1	Activation	[128]
E-box	USF1	Activation	[113]
GATA-box	-	-	[126]
MyoD	-	-	[126]
CCAT-box (Y-box)	p53; Y-box-binding protein	Repression	[133]
Upstream CBF1-binding site	N1ICD Notch1 fragment; CBF1	Activation	[129]

**Table 3:** Multidrug resistance associated with vault overexpression and related mechanisms when known.

Tissue/Cell Lines	Drug(s)	Associated MDR Proteins	Vault Component	Mechanism (When Known)	Reference
Several lung cancer cell lines	DOX	-	MVP, vtRNA	-	[4]
Human colon carcinoma SW620 cells	DOX; vincristine; etoposide; gramicidin D; paclitaxel	-	MVP	-	[142]
U-937 human leukaemia cells	DOX; etoposide; mitoxantrone; 5-fluorouracil	-	MVP *	-	[143]
SW620 human colon cancer cells	Doxorubicin; etoposide; cisplatin; SN-38s	-	MVP *	-	[137]
Ovarian carcinoma	Platinum based; Alkylating	-	MVP	-	[144]
Human lung adenocarcinoma	Cisplatin	Bcl-2; survivin	MVP	-	[138]
Human lung adenocarcinoma A549	Cisplatin	-	MVP	-	[139]
Myeloma cells	Mitoxantrone	-	MVP; vtRNA	-	[4]
Leukaemic cells from AML patients	Mitoxantrone	-	MVP *	-	[145]
GBM	Several, including temozolomide or bis-chloroethylnitrosourea	-	MVP	-	[146]
Breast cancer cell lines cocultured with adipocytes	DOX	-	MVP	Drug efflux from the nucleus	[125]
UMUC-3 human urothelial bladder cancer cell line	DOX	-	MVP	Drug export from the nucleus to lysosomes	[147]
HCC cell lines	Gefitinib (EGFR inhibitor)	-	MVP	Possible uncoupling of AKT activation from EGFR	[82]
Human lung adenocarcinoma	Gefitinib (EGFR inhibitor)	-	MVP	Uncoupling of AKT activation from EGFR	[148]
HCC cell lines	Bleomycin	14-3-3ε	MVP	Drug encapsulation and possible extrusion	[98]
KB nasopharingeal carcinoma cell lines	Cisplatin	-	MVP; vtRNA(s)	Drug efflux from nucleus	[149]
MG63 and U2OS osteosarcoma; U118MG glioblastoma; U-937 lymphoma;	Mitoxantrone	-	vtRNA1-1	Mitoxantrone sequestration by vtRNA1-1	[150,151]

* Detection of increased mRNA levels.
